# South West Orthopaedic Club, Bath

**Published:** 1987-11

**Authors:** 


### South West Orthopaedic Club

### Abstracts of papers given at the Meeting at Bath Royal United Hospital 20th November 1986

### SALTER CHONDROPLASTY

P. May, P. Yeoman, J. Kirkup and P. Morrison
Bath

In 1984 in Toronto Hospital for Sick Children Professor R Salter was employing a containment acetabuloplasty for infants under the age of 18 months undergoing open reduction of the hip. His technique is as yet unreported. One author (PCM) learned the technique and introduced it to Bath. It enables the surgery of CDH to be completed at one sitting before the age which is usually suggested as being necessary for a formal innominate.

We present the preliminary results and experiences of the first 6 cases carried out in Bath over the last 18 months and describe the operative technique in detail. Briefly a formal open reduction and capsular reef is carried but a Salter-Harris I fracture is created to separate the acetabular roof cartilage from the pelvic bone. Its angle is brought to a more correct degree and 3 small bone grafts are inserted to maintain the new position. Postoperatively a modified frog-plaster is applied for 3 months and then a Dennis-brown harness fitted for a further 3 months.

The post operative films are very impressive as the bone graft instantly creates a new and more normal acetabular roof angle. By the time the plaster cast period is over the graft has matured creating a very normal looking acetabulum. No cases of re-dislocation have occurred in this short series. Follow-up is from 6-18 months. One case of mild epiphysitis will be discussed along with the various factors that could have been involved in its cause. Discussion of this technique will be invited.

### THE LONG TERM RESULTS OF HIP SURGERY IN THE SPINA BIFIDA CHILD

H. Weisl, J. Fairclough and G. Jones
Cardiff

At Cardiff, all children suffering from spina bifida who had undergone surgery to their hips were reviewed. A total of 78 patients were assessed, fifty nine at a special review clinic and the remaining nineteen from recent notes and radiographs. There were 45 patients in whom a Sharrard's "Posterior Ilio-Psoas Transfer" had been carried out on 64 hips and 33 patients who had had varus-rotation osteotomy on both hips. The two groups were sequential with the Sharrard's operation (average age at operation 3.7 years) being used exclusively initially but being replaced by the varus osteotomy (average age at operation 6 years) in 1974.

The indications for the two procedures were similar being for subluxation or muscle imbalance of the hips to avoid later dislocation. Both procedures were effective in containing the hips with only 5 (11%) of the Sharrard's patients being dislocated at review and only 2% of the osteotomy group.

There were a large number of surgical complications in both groups, bleeding (11%) and hip stiffness (23%) being more frequent in the Sharrard's patients and spontaneous fracture (38%) and shortening (29%) in the osteotomies.

Review of the walking ability at follow up showed that the percentage of children walking at each age decreased with time. The majority of teenage patients were not walking despite having contained hips.

It is concluded that both posterior ilio-psoas transfer and varus osteotomy are effective in containing the hip in the spina bifida child. However in the long term the walking ability of the teenage spina bifida child is dictated by the initial lesion, the child's and its social environment and not by any one surgical procedure in this multi-factorial disease.

### SCALENECTOMY IN THE TREATMENT OF THORACIC OUTLET COMPRESSION

P. Miller
Royal United Hospital, Bath

A series of patients were studied who we believed to be suffering from thoracic outlet compression due to abnormalities of the scalenus anterior muscle alone.

The 15 patients were selected on clinical grounds and apart from excluding a bony abnormality, other investigations were found to be largely unhelpful.

The vast majority were female and the dominant limb usually affected.

Our patients presented typically with neurological and vascular symptoms, but pain especially with use was the major complaint and pulse diminution the commonest objective physical sign.

Six patients were initially treated by scalenotomy, but half required re-exploration within six months. Dense fibrous tissue was found, binding down the subclavian artery and brachial plexus, requiring total scalenectomy and neurolysis.

Since then a further eleven scalenectomies have yielded excellent results with no recurrent symptoms at six month and three year follow ups.

Particularly successful were a group of young athletic females, where a traumatic aetiology could be postulated.

Other authors have noted this propensity to heal with dense fibrosis after scalenotomy, and the morbidity of more radical procedures. It is our experience that scalenectomy alone is a simple, safe and effective procedure in this group of patients.

### THE DEANE INTERCONDYLAR KNEE

M. W. Regan
The Bath and Wessex Orthopaedic Hospital

The Deane Intercondylar Knee was devised by Mr Graham Deane in the early 1970's. It is a sophisticated multiaxial intercondylar multiaxial intercondylar prosthesis which achieves stability at full extention by its saddle like configuration. On flexion sliding occurs to 40° when a true ball and socket mode allows multiaxial movement between the metal tibial and polyethylene femoral components.

53 patients had 63 Deane knee replacements performed in Bath between 1977 and 1983. (Mean follow-up 7 years).

44 knees are still in situ (70%) and 19 knees have been revised (30%). Of those in situ 30 knees were reviewed clinically and 14 knees were reviewed by questionnaire.

Of those reviewed clinically, 27% had luscent lines on x-ray of more than 1 mm., and 43% had a valgus or varus deformity of more than 10°. Interestingly in this group 83% of patients felt that their knees were satisfactory and 87% felt that their knees were stable. 79% of patients had rheumatoid arthritis.

19 knees were revised. At operation 32% were found to have loose femoral components, 21% loose tibial components and in 47% both components were found to be loose. Marked wear of the femoral component was also a feature of many of the components removed at revision.

In conclusion The Deane Intercondylar Knee offered operative simplicity with minimal bone loss. This certainly made revision surgery much easier. The high failure rate in this series may in part be due to the high percentage of patients with rheumatoid arthritis and the pre-operative severity of their joint disease.

### THE ST GEORG SLEDGE UNICOMPARTMENTAL KNEE ARTHROPLASTY
### A PROSPECTIVE REVIEW OF 115 CASES

**J. G. MacKinnon, S. K. Young and R. A. J. Baily**
**Winford Orthopaedic Hospital, Bristol**

The 'sledge' consists of a biconvex metal femoral runner and a flat UHMWPE tibial surface. It is indicated in arthritic knees with significant disability. Patello-femoral or contralateral tibio-femoral symptoms, instability, excessive varus or valgus or marked fixed flexion are strict contraindications.

115 arthroplasties were performed, the majority in women, medial compartments, and for O.A. The mean age was 69 years and follow-up was from 2–12 years with a mean of 4.5.

The results at two years and last follow-up showed excellent relief of pain and restoration of function. Actuarial analysis demonstrated survivorship of 80% at 12 years.

There were no episodes of infection. Seven tibial components loosened, one disintegrated and four sank or tilted without loosening. One femoral component broke after its owner fell.

We consider the good results were due to:

(1) The non-constrained design of the prosthesis — the only force it transmits is compression. No torque is generated.
(2) Varus/valgus deformity was specifically undercorrected to protect the other tibio-femoral compartment; in series where full correction was applied severe symptoms arose in the newly loaded compartment.
(3) Case selection was strict. Generous indications in other series have increased the number of poor results.

The operation is straightforward and rapid, requiring no special jigs. The prosthesis is relatively inexpensive. In selected patients, the sledge has much to offer.

### HINDFOOT STABILISATION IN RHEUMATOID ARTHRITIS

**A. Simmonds**
**New Zealand**

The paper discusses the management of the rheumatoid hindfoot and in particular stabilisation. A brief discussion of the natural history took place in which our experience of rheumatoid disease was reviewed. While the disease is common in the hindfoot it is relatively uncommon in the ankle joint where secondary valgus or rarely varus deformity of the hindfoot gives rise to secondary biomechanical stresses that contribute to rheumatoid diastasis of the ankle. It is the philosophy of this paper that to stabilise the hindfoot at an early stage in the management of the rheumatoid hindfoot before deformity has taken place prevents the development of severe ankle joint deformity.

The first 10 cases using this philosophy were reviewed and a description of the operative process involving a talo-calcaneal screw fixation and the talo-navicular sliding bone block held in place with a staple together with sub-talar joint in situ bone grafting were described. These patients made a rapid recovery being immobilised for a short period and were back in regular footwear at 12 weeks, the results were promising and it was felt that early stabilisation of the hindfoot could be recommended before deformity took place which proves to be an easy surgical solution.

### CORRECTIVE OSTEOTOMY OF THE LUMBAR SPINE IN ANKYLOSING SPONDYLITIS

**C. H. Marsh, P. M. Yeoman**
**The Bath and Wessex Orthopaedic Hospital**

The progressive spinal deformity seen in patients with advanced ankylosing spondylitis causes disabling postural changes and a loss of visual horizon. Correction of the flexion deformity can be achieved by spinal osteotomy, usually at the lumbar level, and such a procedure has been performed at this centre since 1970.

This paper describes our experience of treating 14 such patients. The technique allows safe controlled correction of up to 60° and no patients have sustained major neurological complications. Secure internal fixation of the osteotomy allows early ambulation thus avoiding prolonged recumbency, and the types of fixation employed and their limitations will be discussed.

The restoration of a vertical posture with horizontal vision has great beneficial effects on the physical and psychological well-being of these patients.

### THE GOOD, THE BAD, AND THE UGLY

**N. A. Purry**
**Medical Officer, Bristol ALAC**

This is a retrospective survey of the first 100 lower limb primary amputation stumps seen at Bristol ALAC in 1985. Sixty-three of these were in men and 37 in women. Their ages on the day of amputation ranged from 0–90 with an average age of 68 years. Sixty-two were aged between 60 and 80 years and there were actually more patients aged over 80 than under 60. Exactly half had peripheral vascular disease and a further 29 amputations were for diabetic gangrene. Ten were for trauma—mainly to motor cyclists. Three were for carcinoma, 2 for congenital limb deformities, 2 for infection, 2 for trophic ulceration, 1 for deformity following poliomyelitis and 1 for varicose ulceration.

Bristol ALAC serves an area from Cheltenham to Salisbury and these patients were referred from 16 different hospitals by 44 different consultants; 47 were referred by 2 hospitals and 31 by 2 surgeons. Twenty-nine consultants only referred 1 patient during a 6 months period. It is appreciated that in many cases the amputation is not done by the consultant but by a member of his team. Patients referral letters were dated between 0 and 141 days after operation with 74 within 20 days of amputation. They first attended the Centre between 10 and 166 days after amputation, 55 within 30 days and 70 within 40.

Criteria for assessing the quality of an amputation stump include whether the wound is healed, whether the stump is conical or bulbous, whether it is optimum length, when it is felt suitable for fabrication of temporary and permanent prostheses, and in the cases of below knee amputation, whether an ischial bearing AK/BK pylon is necessary initially and at what stage it can be cast for a PTB pylon.

The single CHOPART amputation (for congenital deformity) was excellent on presentation, as was one of 2 Symes amputations but the other was not fully healed. There were 52 below knee amputation stumps in this series and it is stressed that patients with an amputation at this level are very much less disabled than those with more proximal amputation. Only 22 (42%) were considered an ideal length (5–5½") at presentation. Twenty-two (42%) were not fully healed. In some cases it appeared that healing had been delayed or skin grafting had been performed in order to preserve an unnecessarily long stump. Twenty-one (40%) were an unsatisfactory shape. In some cases this was due to terminal oedema for which faulty bandaging sometimes appeared responsible but in others it was due to inadequate tapering of the posterior muscle flap in which the residual limb may remain a bad shape for years. Overall 41 below knee stumps (69%) were in some way unsatisfactory but if minor variation in length were accepted the number of entirely satisfactory stumps rose to 15 (29%) and 29 (55%) were considered suitable for PTB pylons when first seen.

One patient had bilateral through knee disarticulations and there were 15 Gritti Stokes amputations in this series. Three of these were not healed on presentation. Amputation at this level produces a strong residual limb but the prostheses available are ugly and unacceptable to many patients.

Twenty-eight amputations had been performed above the knee of which 9 were less than 4 or more than 6" from the joint. Five were unhealed on presentation and another 5 were swollen distally, overall 15 stumps (54%) were unsatisfactory in some way but only 3 (11%) were unsuitable for immediate measurement for a pylon.

Over the whole series of 100 amputation stumps, 30 were good, 56 were bad and 14 were ugly when first seen at the Centre. These findings are similar to those of other surveys in different parts of this country and show no improvement since the report by the British Orthopaedic Association in 1972 quoted recently by Professor McColl.

